# Impact of Metabolic Syndrome on Neuroinflammation and the Blood–Brain Barrier

**DOI:** 10.3389/fnins.2018.00930

**Published:** 2018-12-11

**Authors:** Peter Van Dyken, Baptiste Lacoste

**Affiliations:** ^1^Neuroscience Program, Ottawa Hospital Research Institute, Ottawa, ON, Canada; ^2^Department of Cellular and Molecular Medicine, Faculty of Medicine, University of Ottawa, Ottawa, ON, Canada; ^3^Brain and Mind Research Institute, University of Ottawa, Ottawa, ON, Canada

**Keywords:** blood–brain barrier, neuroinflammation, obesity, diabetes, brain homeostasis

## Abstract

Metabolic syndrome, which includes diabetes and obesity, is one of the most widespread medical conditions. It induces systemic inflammation, causing far reaching effects on the body that are still being uncovered. Neuropathologies triggered by metabolic syndrome often result from increased permeability of the blood–brain-barrier (BBB). The BBB, a system designed to restrict entry of toxins, immune cells, and pathogens to the brain, is vital for proper neuronal function. Local and systemic inflammation induced by obesity or type 2 diabetes mellitus can cause BBB breakdown, decreased removal of waste, and increased infiltration of immune cells. This leads to disruption of glial and neuronal cells, causing hormonal dysregulation, increased immune sensitivity, or cognitive impairment depending on the affected brain region. Inflammatory effects of metabolic syndrome have been linked to neurodegenerative diseases. In this review, we discuss the effects of obesity and diabetes-induced inflammation on the BBB, the roles played by leptin and insulin resistance, as well as BBB changes occurring at the molecular level. We explore signaling pathways including VEGF, HIFs, PKC, Rho/ROCK, eNOS, and miRNAs. Finally, we discuss the broader implications of neural inflammation, including its connection to Alzheimer’s disease, multiple sclerosis, and the gut microbiome.

## Introduction

Metabolic syndrome is one of the most wide-spread diseases in North America, with a prevalence of 34% amongst adults in the United States (Aguilar et al., 2015). It is perhaps the leading avoidable cause of premature death (Hennekens and Andreotti, 2013). Metabolic syndrome is an umbrella term encompassing conditions such as obesity, dyslipidemia, hyperglycemia, and hypertension. These often occur together and result in insensitivity to hormones such as leptin, adiponectin, and insulin (Kaur, 2014). Insulin resistance underlies type 2 diabetes mellitus (T2DM). While the causes of metabolic syndrome are complex, high fat diet (HFD), inactive life styles and genetic predispositions are important risk factors. The components of metabolic syndrome result in wide-ranging effects, many of which impact the central nervous system (CNS) and result in neurodegenerative diseases linked to dysfunction of the BBB. Metabolic syndrome also alters blood pressure and arterial stiffness which, in turn, can damage the BBB. However, given that this review focuses on the effects of inflammation on the BBB, this point will not be discussed.

The BBB is a conserved structure preventing and controlling the passage of most blood components into the central nervous system (CNS) (Andreone et al., 2015; Chow and Gu, 2015; O’Brown et al., 2018). Endothelial cells (ECs) joined together by tight junctions (TJs) form its basic structure (Reese and Karnovsky, 1967), while the basement membrane (BM), pericytes, and astrocytes perform supporting and regulatory functions (Andreone et al., 2015; Daneman and Prat, 2015). The integrity of the BBB is central to neural health, and many autoimmune neurological disorders are associated with its breakdown (Garg and Smith, 2015; Zhao et al., 2015; Chakraborty et al., 2017). BBB opening has been largely linked to inflammation. The maturation and invasion of leukocytes, the release of cytokines, and destruction of targeted cells are effects associated with CNS inflammation that can quickly lead to neuronal damage (Varatharaj and Galea, 2017). Although a sealed BBB normally prevents the passage of immune cells into the CNS, inflammation induces BBB opening by altering its various components (Ransohoff et al., 2015), as we will discuss in this review.

### Components of the BBB

In peripheral organs like the liver or kidneys, capillaries contain fenestrae, little gaps in the vessel wall that allow passage of nutrients, proteins, and even cells into surrounding tissues (Claesson-Welsh, 2015; Augustin and Koh, 2017). In most brain areas, however, ECs are connected by TJs that prevent proteins and cells from crossing the vessel wall. Endothelial TJs are formed by the transcellular proteins claudin, occludin, and junctional adhesion molecules (JAMs) (Daneman and Prat, 2015). Claudins are particularly important for barrier function, as loss of claudins greatly increases barrier permeability (Hartsock and Nelson, 2008). Occludins are highly enriched at the BBB, but they do not appear to be essential to barrier function, as occludin-deficient mice retain normal BBB functioning. Nevertheless, occludins are implicated in calcium flux across the BBB (Hartsock and Nelson, 2008). JAMs, particularly JAM4, regulate passage of leukocytes across the BBB and paracellular permeability. In addition to transcellular proteins, zona occludens-1 (ZO-1), ZO-2, and ZO-3 are important for localizing claudins to the TJs and for connecting them to the cytoskeleton (Hartsock and Nelson, 2008; Daneman and Prat, 2015).

Due to a lack of plasmalemma vesicles, brain ECs have very low rates of transcytosis (Ben-Zvi et al., 2014; Andreone et al., 2017; Chow and Gu, 2017). They display low expression of leukocyte adhesion molecules (LAMs) and of ligand-specific transporters. Passage across the BBB is mostly controlled by these transporter proteins. The luminal and abluminal sides possess different proteomic profiles, allowing the BBB to isolate the vascular and perivascular environments (Daneman and Prat, 2015). Many of these transporters, such as the glucose transporter-1 (GLUT1) are passive, allowing their substrates to flow down their concentration gradients (Zhao et al., 2015). Other transporters consume energy, using ATP hydrolysis to fuel the transport of substrates against their concentration gradient. *P*-glycoprotein, for instance, removes toxins from the brain, including β-amyloid (Aβ), a major player in AD (Deo et al., 2014). This tight control on transport across ECs creates strict regulation and polarization of the vascular and perivascular environments (Chow and Gu, 2015; Daneman and Prat, 2015).

Other structures involved in the BBB include astrocytes, pericytes, and the BM. The latter is made of collagens, laminin, nidogen, heparin, and other glycoproteins. It forms a further barrier in the BBB, but can be disrupted by matrix metalloproteinases (Thomsen et al., 2017). Pericytes are embedded within the endothelial BM and form direct connections with ECs. They are involved in the regulation of angiogenesis and vascular stability, and in controlling the BBB (Armulik et al., 2010; ElAli et al., 2014). Astrocytes are the most abundant glial cell type and play important roles in neurovascular regulation. Their processes ensheathe parenchymal blood vessels with their endfeet. They regulate vasomotor responses and cerebral blood flow in response to changes in neural activity in a given region (Attwell et al., 2010; Petzold and Murthy, 2011; Lacoste and Gu, 2015). They also release factors regulating the maturation and maintenance of the BBB (MacVicar and Newman, 2015; Zhao et al., 2015).

### Inflammation and BBB Disruption

#### CNS Immune Privilege and Leukocyte Invasion

Unlike most organs, the healthy CNS contains very few immune cells, parenchymal microglial cells being the sole population. In the healthy CNS, microglia are maintained without reconstitution from the bone marrow and have no contact with plasma proteins, allowing the CNS to maintain an immunosuppressed environment (Ransohoff and Engelhardt, 2012). The CNS tightly controls, and generally prevents, the passage of immune cells into the perivascular space. This ‘CNS immune privilege’ is largely accomplished by the BBB, which limits leukocyte extravasation (i.e., diapedesis) across the endothelium (Zhao et al., 2015). Undue leukocyte extravasation into neural spaces can result in autoimmune disorders such as multiple sclerosis (MS) (Ransohoff and Engelhardt, 2012).

Leukocyte extravasation requires interactions between adhesion molecules on ECs and leukocytes. LAMs expressed by ECs include P-selectin, E-selectin, intercellular adhesion molecule-1 (ICAM-1) and vascular cell adhesion molecule-1 (VCAM-1) (Su et al., 2012). The selectins bind to P-selectin glycoprotein ligand (PSGL-1), while ICAM-1 and VCAM-1 bind to α4-integrins on leukocytes. After the initial binding event, immune cells roll along the vessel wall releasing chemokines that strengthen their binding interactions. This dramatically reduces cell motility, allowing immune cells to crawl along the vessel in search of a point for extravasation (Engelhardt, 2008; Engelhardt and Ransohoff, 2012). Most transmigration occurs via a paracellular route, which depends on interactions with platelet endothelial cell adhesion molecule (PECAM) and JAM-A (Muller, 2011; Engelhardt and Ransohoff, 2012; Tietz et al., 2018). It can also happen transcellularly, generally when the leukocyte is unable to find an endothelial junction and is strongly activated. The immune cell extends pseudopods and passes right through the endothelium. This phenomenon involves clustering of ICAM-1 and can be inhibited by blocking PECAM. Calveolin-1 plays an important role, especially in the migration of Th1 cells (Lutz et al., 2017). Microtubules are also critical in both types of movement, transporting molecules involved in extravasation to required sites (Muller, 2011).

Leukocyte adhesion molecules are suppressed in the neurovasculature, limiting the opportunity for leukocyte invasion (Chow and Gu, 2015; Daneman and Prat, 2015). The BM forms a second barrier to immune entry. To pass through, leukocytes must be helped by matrix metalloproteinases, such as MMP-9, which clear away membrane filaments. Inflammatory factors open the BBB by upregulating these various factors (Engelhardt and Ransohoff, 2012).

A process called immunosurveillance monitors antigens in the brain and mounts an immune response if foreign bodies are detected. This largely takes place in the cerebrospinal fluid (CSF). Immune cells enter the ventricles through the choroid plexus (CP), a structure in the cerebral ventricles that produces CSF (Ghersi-Egea et al., 2018). There, they are exposed to antigen presenting cells (APC) and drain through meningeal lymphatic vessels into the to the deep cervical lymph node (Louveau et al., 2018). Cells activated in the CSF activate a pro-inflammatory signaling pathway that triggers opening of the BBB and allows leukocyte infiltration, as reviewed elsewhere (Ransohoff and Engelhardt, 2012). Proper functioning of the CP is highly important for neuronal health (Gelb et al., 2018). More recently, a direct route of immune cell infiltration was identified between the bone of the skull and brain meninges by means of microscopic vascular channels (Herisson et al., 2018).

#### Inflammation and the BBB

Inflammation acts through various pathways to affect gene expression. NF-κB, one of the most important factors, is activated by pro-inflammatory cytokines such as TNF-α and IL-1β. Toll-like receptor-4 (TLR4) binds to microbial molecular patterns and activates myeloid differentiation primary response gene 88 (My88), which also leads to NF-κB activation (Lawrence, 2009). The JAK∖STAT pathway is activated by a wide variety of signaling molecules, including interferons (IFNα/β/γ), interleukins (IL-2/3/4/5/6 etc.), and growth factors. These ligands bind to their receptors, leading to activation of STATs which affect gene regulation (O’Shea et al., 2015). Mitogen-activated protein kinase (MAPK) has a role in three separate pathways, including extracellular-signal-regulated kinases (ERKs), Jun amino-terminal kinases (JNK), and p38/stress-activated protein kinases (SAPKs). JNK leads to the activation of c-Jun and c-Fos, which dimerize and form activator protein 1 (AP-1) (Morrison, 2012). Receptors involved in inflammatory responses include cytokine and growth factor receptors, receptor tyrosine kinases, G-coupled protein receptors, and integrins (Morrison, 2012).

Reactive oxygen species are important mediators of inflammation. They are produced as a natural biproduct of aerobic respiration in the mitochondria, through NADPH oxidase activity in phagocytes, and through uncoupled nitric oxide (NO) production (Mittal et al., 2014). Through their oxidative activity, they promote the formation of disulfide bonds between cysteine residues on proteins, affecting protein function (Mittal et al., 2014). In particular, signaling pathways including NF-κB, JNK, and JAK/STAT can be altered by ROS (Nakano et al., 2006; Ray et al., 2012; Hoesel and Schmid, 2013), leading to upregulation of inflammatory cytokines such as transforming growth factor beta (TGF-β), IL-1, IL-6, IL-18, and TNF-α (Elmarakby and Sullivan, 2012). This leads to further inflammation and leukocyte infiltration.

**FIGURE 1:**
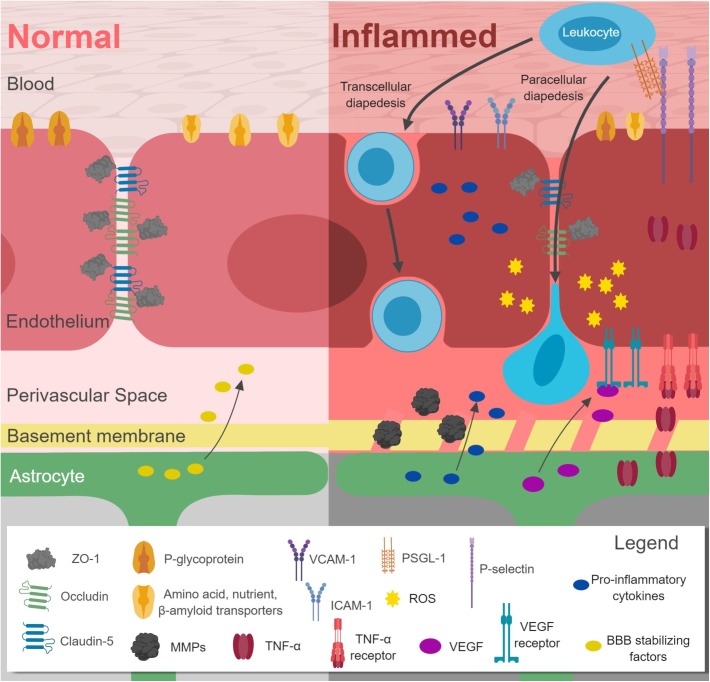
Effects of inflammation on the blood–brain-barrier (BBB). Downregulation of claudin-5, occludin, ZO-1, leptin and amino acid transporters, and P-glycoprotein occurs in conjunction with upregulation of VCAM-1, ICAM-1, P-selectin, MMPs, and pro-inflammatory cytokines. Leukocyte extravasation (diapedesis) increases. Astrocytic gene expression shifts away from BBB stabilizing factors toward VEGF and pro-inflammatory cytokines. The basement membrane is also disrupted. *Figure made with BioRender*.

Increased inflammation leads to disruption of the BBB (Figure 1 and Table 1). TJ proteins, including claudin-5, ZO-1, occludins, and caveolin are downregulated (Chehade et al., 2002; Argaw et al., 2009; Pfeiffer et al., 2011; Sajja et al., 2014; Yoo et al., 2016; Varatharaj and Galea, 2017; Xu et al., 2017). Transcytosis is also altered, together with downregulation of *P*-glycoprotein, leptin, and amino acid transporters (Ransohoff et al., 2015), but upregulation of influx transporters for TNF-α, lysosomal enzymes, Aβ, and monoamines (Varatharaj and Galea, 2017). Fibrin is transported across the disrupted BBB and deposits as insoluble fibrin, a key activator of immune responses (Davalos and Akassoglou, 2012). Interestingly, a recent study successfully targeted fibrin with 5B8 antibody, which inhibited fibrin-induced inflammation without affecting clotting (Ryu et al., 2018). In response to inflammation, leukocyte extravasation increases, with upregulation of VCAM-1 and ICAM-1 (Chai and Song, 2014), as well as P and E-selectin (Carvalho-Tavares et al., 2000). Upregulated MMPs degrade the BM, allowing leukocyte passage (Thomsen et al., 2017). Astrocyte endfeet are disrupted, impairing their ability to maintain BBB integrity (Fan et al., 2014). Astrocyte gene expression shifts toward a pro-inflammatory and cytotoxic state, including production of interleukin-1β (IL-1β), interleukin-6 (IL-6), tumor necrosis factor-alpha (TNF-α), and prostaglandins (Zamanian et al., 2012). TNF-α further promotes inflammatory responses, inducing extravasation of macrophages by acting through NF-κB (Skelly et al., 2013). TNF-α and IL-1β both induce expression of chemokines CXCL1 and CCL2, which are involved in immune cell recruitment to brain ECs. Other pathways become affected by pro-inflammatory states. For example, Wnt/β-catenin is regulated by NF-κB activation, as reviewed elsewhere (Ma and Hottiger, 2016). Inhibition of Wnt/β-catenin leads to increased expression of VCAM and Caveolin-1, proteins critical for transmembrane trafficking (Lengfeld et al., 2017). Overall, inflammation increases BBB permeability, promoting leukocyte extravasation, increasing diffusion of solutes across the BBB, and allowing entry of pathogens and toxins into the CNS. This further stimulates inflammatory responses in a vicious cycle.

**Table 1:** The effects caused by inflammation, obesity, and T2DM on the BBB.

**Effects of inflammation on BBB**	
Downregulation of TJ proteins claudin-5, ZO-1, and occludin	Chehade et al., 2002 ; Argaw et al., 2009; Pfeiffer et al., 2011; Sajja et al., 2014; Yoo et al., 2016; Varatharaj and Galea, 2017; Xu et al., 2017
Downregulation of *P*-glycoprotein and of leptin and amino acid transporters	Ransohoff et al., 2015
Upregulation of influx transporters for TNF-α, lysosomal enzymes, Aβ, monoamines	Varatharaj and Galea, 2017
Transport of fibrin and deposition as insoluble fibers	Davalos and Akassoglou, 2012; Ryu et al., 2018
Upregulation of VCAM-1 and ICAM-1	Chai and Song, 2014
Upregulation of P and E-selectins	Carvalho-Tavares et al., 2000
Upregulation of MMPs	Thomsen et al., 2017
Disruption of astrocytic endfeet	Fan et al., 2014
Shift in astrocytic gene expression toward pro-inflammatory cytokines	Zamanian et al., 2012
Increased extravasation of macrophages	Skelly et al., 2013
Dysregulation of Wnt/β-catenin pathway	Ma and Hottiger, 2016; Lengfeld et al., 2017
**Effects of obesity on BBB**	
Increased activation of NF-κB through TLR4 mediated SFA signaling	Milanski et al., 2009
Upregulation of pro-inflammatory cytokines	Yudkin et al., 1999; De Souza et al., 2005; Zhang et al., 2005; Hommelberg et al., 2009; Lawrence, 2009; Jais and Brüning, 2017
Downregulation of metabolic and housekeeping genes	Ouyang et al., 2014b
Amplified ROS production	Zhang et al., 2005; Tucsek et al., 2014; Jais and Brüning, 2017
Activation of microglia	Tucsek et al., 2014
Infiltration of macrophages into the parenchyma	Stranahan et al., 2016
Insensitivity to anorexic hormones leptin and insulin	Kievit et al., 2006; Zhang et al., 2008
Disruption of the hypothalamus	Prada et al., 2005; Horvath et al., 2010; Jais and Brüning, 2017
Disruption of the hippocampus and cognitive impairment	Kanoski et al., 2010; Davidson et al., 2012, 2013; Hargrave et al., 2016
**Effects of T2DM on BBB**	
Increased ROS production	Wang et al., 2012
Upregulation of pro-inflammatory cytokines	Elmarakby and Sullivan, 2012
Downregulation of TJ proteins	Chehade et al., 2002; Argaw et al., 2009; Sajja et al., 2014; Yoo et al., 2016; Xu et al., 2017
Increased permeability of BBB	Hawkins et al., 2007; Fujihara et al., 2016
Upregulation of VCAM-1 and ICAM-1	Janelidze et al., 2017
Thickening of the BM	Junker et al., 1985
Increased MMP activity	Hawkins et al., 2007; Thomsen et al., 2017
Increased levels of AGEs	Kowluru et al., 2004

Conditions that increase systemic inflammation impacts many of the systems in the body, including the brain. As such, inflammation is increasingly being implicated in neuropathology, as we will explain below.

## Obesity and the BBB

Obesity is the excess accumulation of body fat caused by an imbalance between energy intake and consumption. It results in high levels of adipose tissue, a hormonally active tissue that secretes adipokines and cytokines. As the number of adipose cells increase, more of these regulating proteins are secreted, causing tissues to develop resistance to their effects. For example, leptin resistance prevents proper satiety so that hunger still occurs despite excess fat. Insulin resistance prevents proper intake and use of blood glucose, causing hyperglycemia. These hormones, and their disruption, affect nearly every organ in the body, making obesity a contributing factor in many diseases, including cardiovascular disease, fatty lipid disease, and neurological disorders like AD (Blüher, 2013).

### Obesity and Cerebral Inflammation

**FIGURE 2:**
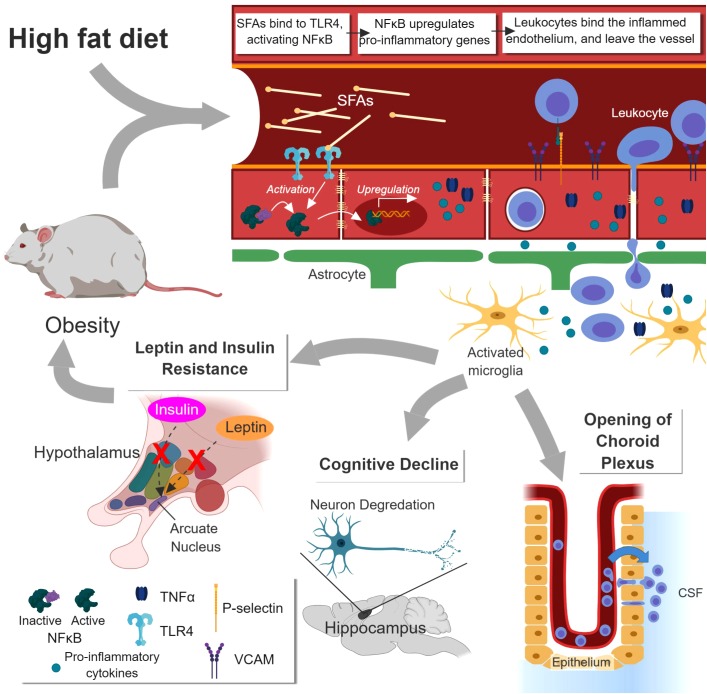
Effects of obesity on the BBB and the brain. Increased saturated fatty acids (SFA) concentration caused by high fat diet (HFD) or obesity enhance NF-κB-mediated inflammation at the BBB via TLR4 receptor. This causes increased leukocyte extravasation, release of pro-inflammatory cytokines, and activation of microglia. The hypothalamus is disrupted, leading to leptin and insulin insensitivity and greater obesity. Disruption of the hippocampus leads to cognitive impairment. Opening of the choroid plexus leads to enhanced leukocyte influx into the cerebrospinal fluid (CSF), more antigen sampling, and greater risk of immune response. *Figure made with BioRender*.

The effects of obesity are largely mediated through inflammation (Figure 2). High fat diet (HFD) is commonly used to study obesity in mice, and neural inflammation can be seen even before significant weight gain in these murine models. Saturated fatty acids (SFAs), like palmitate, play an important role in activating these early inflammatory pathways. SFAs interact with toll-like receptor 4 (TLR4) and activate myeloid differentiation primary response gene 88 (MyD88) (Milanski et al., 2009), which leads to activation of NF-κB. This pathway is linked to inhibition of anorexic hormones insulin and leptin, partially through increased expression of NF-κB-induced expression of suppressor of cytokine signaling 3 (Kievit et al., 2006; Zhang et al., 2008). NF-κB also upregulates pro-inflammatory cytokines including Il-1β, TNF-α, and IL-6 (Yudkin et al., 1999; De Souza et al., 2005; Zhang et al., 2005; Hommelberg et al., 2009; Lawrence, 2009; Jais and Brüning, 2017), causing a decrease in TJ protein expression and an decrease in BBB integrity (Gustafson et al., 2007). Metabolic, housekeeping, and structural genes are downregulated in mice with diet-induced obesity (Ouyang et al., 2014b). Amplified ROS production results from increased mitochondrial respiration, upregulated expression of NADPH oxidase, and the action of inflammatory cytokines (Zhang et al., 2005; Tucsek et al., 2014; Jais and Brüning, 2017). They further promote cytokine expression and oxidative stress (De Souza et al., 2005; Dorfman and Thaler, 2015). Microglia are activated and upregulate their Fcγ receptors, priming them for response to IgGs (Tucsek et al., 2014). Immune cells are also activated, and macrophages infiltrate the brain parenchyma (Stranahan et al., 2016).

One of the earliest regions to experience HFD-induced inflammation is the hypothalamus (Prada et al., 2005). As a major control center for energy and weight balance, its impairment contributes to the development of obesity (Jais and Brüning, 2017). Hypothalamic inflammation precedes weight gain by a few days in mice on HFD (Thaler et al., 2012). As described above, SFAs promotes leptin and insulin resistance through the NF-κB pathway, impairing the hypothalamus’ ability to lower hunger and regulate blood sugar (Boden and Shulman, 2002; Jais and Brüning, 2017). Long term HFD reduces the number of synapses on hypothalamic neurons and increases neural apoptosis (Horvath et al., 2010).

High fat diet also leads to cognitive deficits (Pistell et al., 2010). Several studies by Davidson et al. (2012, 2013) have looked at the impact of HFD on the hippocampus, a major center for learning and memory (Kanoski et al., 2010; Hargrave et al., 2016). In one, mice were challenged to determine if they could associate external landmarks with a food reward, an operation dependent on the hippocampus. Mice on HFD were less able to make the association compared with controls fed on a regular diet (Hargrave et al., 2016). Further experiments tested their ability to discriminate between similar stimuli. While mice with diet-induced obesity performed as well as the control in the hippocampus-independent test, they performed poorly on the hippocampus-dependent discrimination test (Davidson et al., 2012, 2013). Interestingly, cognitive impairment occurred before obesity developed, and while it was a good predictor of obesity, the latter was a poor predictor of impairment. Even further, cognitive deficits could be identified before significant BBB leakiness occurred (Davidson et al., 2013). This raises questions about the development and time course of BBB pathology. Cognitive deficits and corresponding BBB disruption can be detected as early as 24 days after HFD (Davidson et al., 2013). However, plasma triglyceride and free fatty acid levels do not appear significantly altered that early. There are a number of possible contributing factors. Cognitive deficits were associated with elevated blood glucose levels, and obese rats show decreased GLUT1, a deficiency correlated to impaired cognitive performance (De Vivo et al., 1991). Insulin resistance may also play a role (Hargrave et al., 2016). Indeed, insulin resistance of brain ECs is known as type III diabetes, and it is strongly associated with AD. It is possible that increased Aβ levels may play a role, as this is caused by increased levels of circulating fats and is associated with BBB disruption (Hargrave et al., 2016). None of these factors, however, have been definitively linked with early BBB decline in response to HFD.

Many studies on BBB integrity have looked at regulatory regions like the hypothalamus or memory and learning regions like the hippocampus. A few, however, have looked at the CP, a structure found in the ventricles of the brain that produces the CSF. While it doesn’t possess a tight barrier, the CP largely controls the flow of immune cells into the CSF, hence playing a major role in brain immunosurveillance (Schulz and Engelhardt, 2005; Engelhardt, 2006; Ransohoff and Engelhardt, 2012; Ghersi-Egea et al., 2018). In one study, rats exposed to HFD showed a decrease in TJ integrity and corresponding increase in CP leakiness (Kanoski et al., 2010). Another study showed decreased passage of insulin-like growth factor-1 (IGF-1), a neuroprotective factor related to neuropathologies, across the CP barrier (Dietrich et al., 2007). These point to a direct link between neuroimmune disorders and HFDs.

### Obesity, Leptin, and Cerebral Inflammation

Many obesity-associated pathologies result from increased production of adipocytes. These, in addition to being the body’s predominant fat storage cell, secrete a number of signaling molecules called adipokines. One of these, leptin, is produced by adipose tissue when the body’s energy needs are met, acting on the hypothalamus to increase satiety. An increase in adipocyte number leads to higher secretion of leptin, however, despite the high plasma concentration of leptin in obese individuals, they continue to feel hunger. Indeed, the brain develops a leptin resistance, preventing the hormone from exerting its usual effect. Leptin resistance, and factors causing it, has been reviewed elsewhere (Sáinz et al., 2015).

One of the primary possible causes for leptin resistance is ineffective transport across the BBB. Leptin receptors at the BBB are responsible for modulating leptin transport into the parenchyma [although they do not necessarily transport it (Banks and Lebel, 2002)], and they seem to be easily saturated. Obese mice have higher plasma levels of leptin than WT counterparts, but similar levels of leptin in the CSF, suggesting that leptin transport, while not diminished by obesity, does not increase with higher plasma concentrations of leptin (Burguera et al., 2000; Nave et al., 2003). Administering leptin into the plasma of an obese mouse has no effect, but delivering it directly to the brain produces a robust leptin response (Van Heek et al., 1997). Further research, however, has shown that leptin transport can be reduced in obesity. Mice treated with triglycerides in milk displayed a 44% decrease in leptin transport across the BBB compared to mice given fat-free milk (Banks et al., 2004). Increased plasma triglyceride levels are a key feature of obesity (Musunuru, 2010) and would play an important role in leptin resistance.

Leptin has been implicated in many roles beyond signaling satiety, including modulation of immunity. For example, endothelial leptin signaling plays a role in leukocyte extravasation in the spinal cord and other CNS areas. Knocking out leptin receptors prevents this migration, preserves TJ integrity, and attenuates the progression of experimental autoimmune encephalomyelitis (EAE), a mouse model of MS (Lang and Ratke, 2009). Leptin also acts directly on immune cell populations. It promotes the development and activation of Th1 leukocytes, cells associated with the development of EAE (Lord et al., 1998; Matarese et al., 2001b). Conversely, the Th2 immune response, which plays a protective role in EAE, is diminished by leptin (Zamvil and Steinman, 1990; Matarese et al., 2001b). Increased leptin also correlates with a decreased number of Tregs, a regulatory T cell that inhibits inflammation and immune response (Matarese et al., 2005; De Rosa et al., 2007).

These effects of leptin on immune cells are at least partially mediated by the mTOR signaling pathway (Galgani et al., 2010; Procaccini et al., 2012). mTOR-mediated leptin signaling leads to a reduction in Treg production, and Tregs will themselves release leptin to control their own responsiveness (De Rosa et al., 2007; Procaccini et al., 2010). mTOR activation also enhances the development and survival of CD4+CD25- (non-regulatory, helper) T cells by upregulating Bcl-5, a factor involved in T cell differentiation (Galgani et al., 2010). This induced shift toward a pro-inflammatory environment has implicated leptin as an essential player in the development of EAE. Indeed, EAE will not develop in leptin deficient mice (Matarese et al., 2001a; Ouyang et al., 2014a). This also accounts for evidence suggesting increased risk of contracting MS amongst obese adolescents (Hedström et al., 2016). On the other hand, leptin acts on astrocyte leptin receptors to enhance beneficial EAE immune response and decrease disease severity (Mishra et al., 2013). Thus, leptin has a multifaceted role in neuroinflammation, the details of which remain to clarified.

## Type 2 Diabetes

An important consequence of obesity is type 2 diabetes mellitus (T2DM). Caused by loss of insulin sensitivity in adipocytes, muscles, and other insulin-dependent cells, T2DM results in a loss of effective glucose control (Pandey et al., 2015). If patients do not regulate their sugar levels through diet and/or exercise, they easily become hyperglycemic. This results in increased inflammation, perturbed metabolic pathways, and a number of complications including retinopathy, nephropathy, neuropathy, and degradation of the BBB (Ennis and Keep, 2007; Campos, 2012; Blake and Trounce, 2014; Mapanga and Essop, 2016).

### T2DM and Inflammation

**FIGURE 3:**
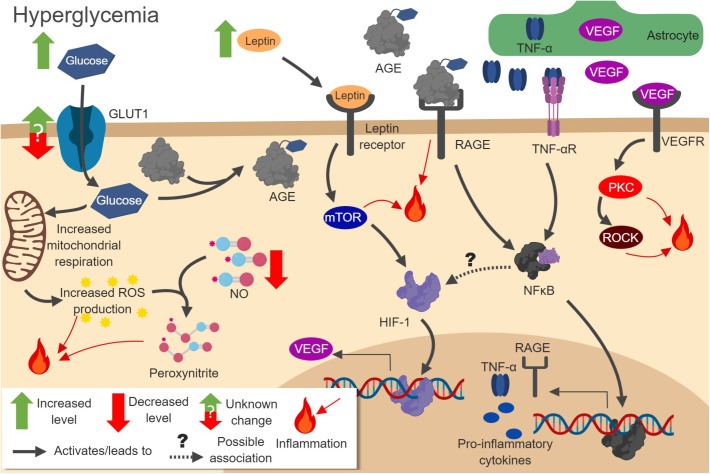
Pathways involved in hyperglycemia-induced neuroinflammation. Increased glucose concentration in cells leads to amplified oxidative respiration and reactive oxygen species (ROS) production. ROS react with NO to produce peroxynitrite. Increased glucose also leads to formation of advanced glycation end products (AGEs), which act on RAGE to increase NF-κB activation. Activated NF-κB increases pro-inflammatory gene expression, including RAGE itself and cytokines. Increased leptin leads to mTOR and HIF-1 pathway activation, increasing vascular endothelial growth factor (VEGF) production. VEGF released from astrocytes activates protein kinase C (PKC) and Rho-associated kinase (ROCK), which further promotes inflammation. *Figure made with BioRender*.

As in HFD models of obesity, T2DM mediates many of its effects through inflammation (Brownlee, 2005) (Figure 3). The hyperglycemia caused by T2DM leads to increased mitochondrial respiration in ECs, pericytes, and astrocytes. This promotes ROS production and oxidative stress. Enhanced ROS activate NF-κB, activator protein-1 (AP-1), and STAT pathways (Wang et al., 2012). This, in turn, leads to upregulation of inflammatory cytokines (Elmarakby and Sullivan, 2012). ROS also interfere with astrocyte communication by inhibiting the folding of gap junction proteins. Damage to astrocytes can be rescued using antioxidants and chaperone proteins (Gandhi et al., 2010). The damage caused by ROS also eventually leads to pericytes loss, BBB breakdown, and astrocytic endfeet degeneration (Brownlee, 2005). Left untreated, this inflammation can lead to cognitive impairment, just as observed in obesity models (Luchsinger et al., 2007).

The impact of T2DM-induced inflammation in the BBB is readily measurable at the molecular level. T2DM downregulates TJ proteins including claudin-5, ZO-1, occludin, and caveolin (Chehade et al., 2002; Argaw et al., 2009; Sajja et al., 2014; Yoo et al., 2016; Xu et al., 2017). This allows greater influx of blood components into the perivascular space, as shown by studies on albumin and (14)C sucrose penetration (Hawkins et al., 2007; Fujihara et al., 2016). CAMs, including ICAM-1 and VCAM-1, are upregulated (Janelidze et al., 2017). The BM also becomes thicker (Junker et al., 1985), an effect associated with angiopathy leading to increased vascular permeability (Roy et al., 2015). BM thickening is promoted by activation of PKC, AGEs, and growth factors such as TGF-β and connective tissue growth factor. It leads to a compositional change in the extracellular matrix. The upregulation of fibronectin, collagen IV, and laminin compromises cell attachment to the BM and BBB permeability, while downregulation of heparin sulfate proteoglycans removes anionic protein binding sites, destabilizing the BM (Roy et al., 2010; Chronopoulos et al., 2011). MMP activity also increases, and while its protease activity does not offset the increased BM synthesis, it is important for leukocyte extravasation (Hawkins et al., 2007; Thomsen et al., 2017).

The rate at which inflammation causes damage is brain region-dependent. A longitudinal study by Huber et al. (2006) found that increased BBB permeability first occurred in the midbrain, 28 days after T2DM was induced. This was followed by the hippocampus, the cerebral cortex, and the basal ganglia (Huber et al., 2006). Whole brain MRI scans found most brain atrophy in the hippocampus and the temporal, frontal, and limbic gray matter. Damage was also seen in frontal and temporal white matter, but to a lesser extent (Moran et al., 2013). Interestingly, damage to the microvasculature continues even after the hyperglycemia is controlled (Huber et al., 2006). This is partially due to the accumulation of advanced glycation end products (AGEs), which have detrimental effects on the brain (Ceriello, 2012).

### Inflammatory Mediators in T2DM

#### Hyperglycemia and Insulin

Pathways mediating the effects of hyperglycemia are complex and not fully understood. Most seem to be an immediate result of increased glucose levels. Unlike many organs, the brain endothelium uses GLUT1 as glucose transporter, which is not directly regulated by insulin (Shah et al., 2012). In theory, hyperglycemia would lead to increased glucose absorption by the BBB. Whether this actually happens is a matter of debate. Much of the early evidence suggested that hyperglycemia lowered GLUT1 (*Slc2a1*) expression, normalizing brain glucose transport (Pardridge et al., 1990; Cornford et al., 1995). However, other research suggested that there was no difference in glucose transport between normoglycemic and hyperglycemic rats (Pelligrino et al., 1992; Jacob et al., 2002). The answer seems to depend largely on the conditions, models, and brain region. For example, one study found downregulated GLUT1 in the retina, but not in the cerebral cortex (Badr et al., 2000). Another found decreased GLUT1 expression in chronic, but not acute, hyperglycemia (Duelli et al., 2000). This controversy has been reviewed in depth (Shah et al., 2012; Prasad et al., 2014). A study by Jais et al. (2016) found that GLUT1 production and glucose uptake decreased in mice on a HFD. However, the resulting inflammation caused upregulation of VEGF, which led to recovery of GLUT1 levels (Jais et al., 2016). This opens new doors for the study of signaling processes involved in BBB response to hyperglycemia.

Perhaps more important than glucose transport is brain glucose concentration. Here, the preponderance of evidence shows increased glucose uptake through the BBB (Shah et al., 2012). This higher glucose concentration and metabolic rate explains the inflammatory phenotype described above. Note that this has no bearing on the GLUT1 debate. While GLUT1 is the most important glucose transporter, there are many others (GLUT3, GLUT4, GLUT8, and SGLT1) that can modulate neural glucose levels. Few studies have been done on human glucose levels, and their results are mixed, suggesting either no change or an increase in glucose concentration (Kreis and Ross, 1992; Seaquist et al., 2005). Some of these have been done on T1DM patients, but not T2DM (Shah et al., 2012). More work is needed to address this gap.

Surprisingly, studies suggest that altered insulin signaling does not play a critical role in type 2 diabetic pathology. For example, in T1DM, where pancreatic insulin production has been cut off and insulin levels are very low, hyperglycemic patients display the same oxidative stress as their type 2 counterparts (Garg et al., 2014; Yorulmaz et al., 2015). Cell transport and permeability are similarly disrupted in each type. Mice lacking brain endothelial insulin receptors maintain normal BBB function and permeability (Kondo et al., 2004). T2DM patients, on the other hand, display insulin resistance. Thus, despite higher insulin levels, patients with type 2 diabetes show little reactivity to insulin, making them functionally equivalent to patients with type 1 diabetes.

#### Role of VEGF

The effects of hyperglycemia involve stimulation of *vascular endothelial growth factor* (VEGF), a factor classically associated with both angiogenesis and vascular permeability.

Vascular endothelial growth factor stimulates vasculogenesis, resulting in immature, unstable vessels. Signaling molecules such as Angiopoietin-1 promote vessel maturation and stabilization (Yancopoulos et al., 2000). VEGF also regulates angiogenesis in response to hypoxia via hypoxia-induced factors (Masoud and Li, 2015).

Because VEGF increases BBB permeability, it is associated with inflammation. For example, chronic intracerebral infusion of VEGF in mice was found to upregulate ICAM-1 and major histocompatibility complex I and II, increasing the opportunity for immune response (Proescholdt et al., 1999). Treatment with VEGF also disrupts claudin-5, occludin, and ZO-1, important TJ proteins (Fischer et al., 2002; Argaw et al., 2009). When given after stroke, VEGF triggers rapid (within minutes) stimulation of caveolae-mediated transcytosis, increasing transcellular transport and permeability (Feng et al., 1999; Chen et al., 2002).

Studies of VEGF expression in T2DM have focused on diabetic retinopathy. Increased VEGF production was found in response to hyperglycemia, an effect linked to the increased retinal vascularization (Cai et al., 2014; Capitão and Soares, 2016; Caprnda et al., 2017). The effects of hyperglycemia on VEGF expression in the rest of the brain are less known. One study found that cultured astrocytes exposed to higher glucose had increased VEGF expression (Wang et al., 2012). Astrocytic VEGF is released directly into the parenchyma, allowing it to act on the abluminal VEGF receptors of the endothelium.

The effects of plasma VEGF depend to a certain extent on the state of the BBB. VEGF receptors are located on the abluminal side of blood vessels, so VEGF must be able to cross the barrier in order to exert its effects (Qu-Hong et al., 1995). Since the BBB is more permeable in diabetic conditions, the pro-angiogenic and vascular permeability effects of VEGF are most clearly observed in those conditions. A study found that inhibiting early VEGF release from ECs could promote stroke recovery in diabetic mice (Reeson et al., 2015). Interestingly, the most commonly prescribed drug for acute management of stroke, rtPA, generates adverse side effects including increased BBB permeability (Suzuki et al., 2016), in great part via activation of VEGF signaling and increased transcytosis (Benchenane et al., 2005; Turner and Vink, 2012).

One of the upstream players regulating VEGF is HIF-1, a transcriptional factor normally upregulated in hypoxic conditions, which increases oxygen supply, glucose transport, and blood vessel formation (Fischer et al., 2002; Argaw et al., 2009; Balamurugan, 2016). It is also upregulated in response to hyperglycemia and to the resulting advanced glycation endproducts (AGEs) and ROS (Treins et al., 2001). Inhibiting HIF-1 activity reduced the hyperglycemic increase in BBB permeability and decreased expression of VEGF (Yan et al., 2012). Mechanisms by which hyperglycemia can dysregulate HIF-1 are reviewed elsewhere (Catrina, 2014). Further upstream, mTOR regulates HIF-1 activity. Inhibiting mTOR lessens the severity of BBB disruption and decreases the upregulation of both HIF-1 and VEGF (Land and Tee, 2007; Wei et al., 2016). As discussed previously, mTOR shifts the immune system away from Tregs and stabilizes Th1 and Th2 cell, priming the immune system for reaction (Matarese et al., 2001b). mTOR is responsive to nutritional status and blood sugar levels. HFD is linked to higher mTOR activity, which is associated with a more active immune system and higher rates of autoimmunity. mTOR is regulated by leptin which, like in obesity, is upregulated in diabetes due to higher levels of fat and blood glucose (Galgani et al., 2010; Bandaru and Shankar, 2011). NF-κB has also been suggested to mediate TNF-α stimulated upregulation of HIF-1 in muscle cells (Remels et al., 2015). This connection remains to be elucidated in the brain context.

Downstream, VEGF activates PKC, enzyme involved in endothelial permeability (Fleegal et al., 2005; Titchenell et al., 2012; Shao and Bayraktutan, 2013). PKC-β plays an especially important role, and hyperglycemia promotes dysfunction in its pathway, causing an increase in NADPH oxidase and MMP-2, and a decrease in occludin (Shao and Bayraktutan, 2013). PKC-β regulates the Rho-kinase (ROCK)/myosin light chain kinase (MLCK) pathway, which plays an important role in vascular smooth muscle tone. ROCK signaling inhibits expression of endothelial nitric oxide synthase (eNOS), which reduces the availability of NO, an effect associated with endothelial dysfunction. ROCK also inhibits eNOS phosphorylation and its resulting activation by inhibiting Akt activity (protein kinase B), as reviewed elsewhere (Yao et al., 2010). Pathological RhoA/ROCK activation in ECs also promotes an association between caveolin-1 and eNOS and their translocation to membrane caveolae compartments (Zhu et al., 2003, 2013). There, eNOS is inhibited (Ju et al., 1997; Drab et al., 2001; Ming et al., 2002), impairing BBB permeability (Siddiqui et al., 2011). Pathological activation of ROCK also promotes oxidative stress (Rivera et al., 2007; Soliman et al., 2012). Pharmacological blockade of ROCK reduces hyperpermeability via inhibition of oxidative stress in ECs (Allen et al., 2010; Gibson et al., 2014). Interestingly, it has been recently demonstrated that genetic or pharmacological blockage of ROCK prevents BBB breakdown following brain injury, opening new possibilities for treatment (Sadeghian et al., 2018).

#### Role of eNOS and NO

Nitric oxide (NO) plays autocrine and paracrine roles in the blood stream. It is synthesized by nitric oxide synthases (NOS), enzymes including neuronal NOS (nNOS), inducible NOS (iNOS), and eNOS. The latter constitutively releases a small amount of NO from ECs, which maintains smooth muscle tone (Kumar et al., 2017). It is also implicated in BBB permeability modulation and angiogenesis (Murohara et al., 1998). eNOS is activated by phosphorylation of serine 1177 in humans. This is modulated by VEGF signaling through a number of pathways. VEGF activates phospholipase C, which induces IP_3_ synthesis and release of Ca^2+^ into the cytoplasm, followed by activation of calmodulin. This pathway leads to activation of PKC, AMP-dependent kinase (AMPK), and Akt (protein kinase B). VEGF can also activate Akt through PI3K. PKC, Akt, and AMPK can all phosphorylate eNOS.

Depending on the model of inflammation used, eNOS has been found to be up or down-regulated. Inflammatory and stroke models show overexpression of NO leading to detrimental effects (Murohara et al., 1998; Mohammadi et al., 2011). Diabetic models, however, show decreased eNOS expression (Safiah Mokhtar et al., 2013). This partly involves ROCK signaling, but control of eNOS is very complex. For example, insulin activates eNOS through the PI3K/Akt pathway. In hyperglycemia, however, the high amount of glucose increases the activity of the hexosamine pathway, which increases the concentration of UDP-β-*N*-acetylglucosamine (UDP-GlcNAc). This factor glycosylates members of the Pl3K/Akt pathway, limiting eNOS activation and subsequent NO activity (Patti et al., 1999; Federici et al., 2002). AGEs can also limit the production of NO by modifying gene expression, glycosylating eNOS, or quenching NO (Xu et al., 2003; Rojas et al., 2010). This creates a strong case for lowered eNOS activity in diabetes.

Nitric oxide has a half-life of a only few seconds but can diffuse freely through the cytoplasm and cell membranes, making it an effective signaling molecule for the local environment (Pacher et al., 2007). NO directly activates cGMP production, which decreases both P-selectin in endothelium and its ligand, β-2 adhesion molecule, in neutrophils. eNOS null mice show persistent upregulation in inflammatory pathways, with 9-10 times more leukocytes binding to the endothelium following an inflammatory stimulus (Cirino et al., 2003). eNOS is also associated with downregulation of MMPs (Tronc et al., 2000).

Nitric oxide plays an inflammatory role in an oxidative environment. It reacts with superoxide to form peroxynitrite, a highly reactive oxidant responsible for many detrimental effects, including disruption of DNA and protein structure (Pacher et al., 2007). Many studies have implicated peroxynitrite as a key player in BBB degradation (Mayhan, 2000; Tan et al., 2004; Parathath et al., 2006; Phares et al., 2007). The peroxynitrite-dependent IFN-γ pathway is sufficient to increase BBB permeability (Phares et al., 2007), and blocking peroxynitrite or superoxide ameliorates inflammation-induced BBB disruption (Mayhan, 2000; Tan et al., 2004). Peroxynitrite also activates latent MMPs (Rajagopalan et al., 1996) and inhibits the MMP inhibitor, TIMP (Frears et al., 1996), causing degradation of the BM.

The role of NO is still somewhat unclear. One study found inactivation of eNOS attenuated the downregulation of claudin-5 and occludin and decreased the BBB permeability caused by VEGF signaling from reactive astrocytes (Argaw et al., 2012). However, this report did not specify whether peroxynitrite was involved. The same can be said for studies that connected eNOS activation with increased BBB permeability in connection with VEGF activation or induced stroke (Fukumura et al., 2001; Mohammadi et al., 2011). Clarification of the precise mechanism responsible for these effects is necessary to evaluate their significance. In addition, peroxynitrite has a multifaceted role, with one study suggesting it protected neurons against nitric-oxide mediated apoptosis (García-Nogales et al., 2003). Thus, more work is needed to elucidate the role of NO in inflammation-induced BBB breakdown.

#### Role of AGEs

AGEs, primary mediators of diabetes-associated pathologies, result from hyperglycemia. Glucose combines with a protein to form a Schiff base that undergoes further modification to become an AGE. AGEs alter protein structure and function, affecting enzyme activity, reducing protein half-life and weakening ligand binding (Vlassara and Palace, 2002). Certain AGEs can cross-link proteins, creating matrixes that inhibit normal protein function. Their production leads to ROS formation that cause damage to the cell by oxidative stress. DNA and lipids are vulnerable to AGE formation. For review see (Ahmed, 2005; Ceriello, 2012).

AGEs act on a variety of receptors, the most common of which is the receptor for AGE (RAGE). RAGE activates an immune response by modulating a number of cytokines, including pro-inflammatory cytokines IL-8, TGF-β1, TNF-α, interferon γ (IFN-γ), IL-2, and IL4, and basement membrane modifying proteins COL-1, COL-III, and MMP-2 (Shimizu et al., 2013; Serban et al., 2016). Many of RAGE’s effects are mediated through NF-κB (Ahmed, 2005). NF-κB activation leads to increased expression of IL-6, IL-1α, and TNF-α. It promotes coagulation and vasoconstriction through the upregulation of tissue factor, thrombomodulin, and endothelin-1. It also increases VCAM-1 and ICAM-1 expression, allowing for enhanced penetration of leukocytes (Lawrence, 2009; Hoesel and Schmid, 2013). Perhaps most importantly, NF-κB promotes further expression of RAGE, creating a positive feedback loop. This has implicated RAGE in metabolic memory, a phenomenon in which oxidative damage and inflammatory gene expression continue even after hyperglycemia has been normalized (Ceriello, 2012). In one study, as little as 2 months of hyperglycemia before glucose control was re-established was enough to cause permanent changes to NF-κB activity (Kowluru et al., 2004). Hyperglycemia greatly alters gene expression, and many oxidation controlling genes are downregulated. These modifications may persist after glucose levels are normalized, further contributing to metabolic memory (Ceriello, 2012). As a result, the inflammatory state induced by hyperglycemia can persist long after blood glucose levels are controlled, increasing the severity of hyperglycemia.

#### Role of miRNAs

Micro RNAs (miRNAs) have also been shown to play a role in BBB disruption. miRNAs are short, 15–20 nucleotide-long RNAs that bind to messenger RNAs, preventing their translation or marking them for degradation (Hammond, 2015). They are increasingly recognized to play an important role in many pathologies, including inflammatory diseases. Pro-inflammatory cytokines TNFα and IFNγ have been shown to downregulate several miRNAs. One of them, miR-125a-5p, regulates barrier tightness and prevents leukocyte extravasation (Reijerkerk et al., 2013). Another, miR-143, is associated with T2DB. It downregulates oxysterol-binding protein-related protein 8, impairing the ability of insulin to induce Akt activation. This creates a miRNA-linked mechanism for insulin insensitivity (Li et al., 2018). miR-143 also downregulates PUMA, a pro-apoptotic molecule. PUMA is associated with BBB damage and permeability, and it decreases TJ protein expression (Bai et al., 2016). These studies open a new mechanistic approach to inflammation, necessitating further investigation.

## Impact of Metabolic Syndrome on Neural Health

### Connections to Aging

The inflammatory effects of metabolic syndrome described above are, in many respects, comparable to effects of aging. While aging is a very complex process not well understood to date, its features appear to be the products of inflammation caused by cell senescence and deterioration, with the accumulation of AGEs and other harmful waste products (Lucke-Wold et al., 2014). Metabolic syndrome often accompanies the aging process, with 46.7% prevalence amongst those over 60 compared to 18.3% amongst those aged 20–39 (Aguilar et al., 2015). It leads to less healthy aging and poorer quality of life. Hypertension and cardiovascular disease have long been known to increase risk of ischemic stroke, and obesity and diabetes are also known risk factors (Lucke-Wold et al., 2014). Metabolic syndrome is also associated with reduced cognitive performance and exacerbates the risk and outcomes of neurodegenerative disease, such as AD and MS (Hassing et al., 2009).

Both obesity and diabetes, through the mechanisms described above, lead to increased neuroinflammation, essentially accelerating the aging process. For example, AGEs produced because of diabetes contributes to production of ROS which causes protein and DNA damage, cell death, and the activation of immune pathways (Ahmed, 2005). High concentrations of leptin induced by obesity lead to immune cell activation (Lord et al., 1998; Matarese et al., 2001b). The damage caused to endothelial cells by increased inflammation and ROS are more susceptible to binding platelets and forming clots (McEwen, 2014).

Astrocytes succumb to the pressures of oxidative stress and telomeric replication exhaustion, becoming unable to meet their role of maintaining ion and neurotransmitter homeostasis, and the BBB (Bhat et al., 2012). The BBB begins to decay, with occludin and ZO-1 significantly reduced (Mooradian et al., 2003), and endothelial cell structure becoming less robust (Lee et al., 2012). Meanwhile, inflammatory pathways are increasingly active. In their review on aging and metabolic syndrome, Lucke-Wold et al. (2014) described a positive feedback loop in which activated microglia produce ROS, leading to cell death and increased local glutamate levels. This, in turn, leads to increased secretion of pro-inflammatory cytokines and further microglial activation. This process would be interrupted in a younger brain by astrocytes buffering excess glutamate (Lucke-Wold et al., 2014).

### Connections to Alzheimer’s Disease

Neuroinflammation is implicated in many neurological disorders, and inflammation caused by metabolic syndrome increases the likelihood and severity of these pathologies. The link between obesity, diabetes and AD is now well established and has been studied extensively (Nguyen et al., 2014; Pugazhenthi et al., 2017). Both obesity and diabetes can lead to insulin resistance in the CNS, a disorder known as type 3 diabetes (de la Monte and Wands, 2008). The resulting lack of insulin and insulin-like growth factors signaling is identical to that seen in AD. Both lead to neuronal death and glial activation. They also result in mitochondrial dysfunction, oxidative stress, and increased ROS levels. The inflammasome, a protein complex leading to pro-inflammatory cytokine secretion, caspase-1, and proteolytic cleavage, gets upregulated and increases inflammation (Pugazhenthi et al., 2017). As described above, this leads to degradation of the BBB and the influx of leukocytes. Aβ formation is upregulated in AD, and the damaged BBB is less able to remove it, causing accumulation in the parenchyma (Bell and Zlokovic, 2009; Zlokovic, 2011; Sagare et al., 2012). In addition, leptin has been implicated in AD pathogenesis. It decreases Aβ production by blocking β-secretase and increasing Aβ uptake, and it deactivates glycogen synthase kinase beta, the protein primarily responsible for tau hyperphosphorylation (Folch et al., 2015). Although leptin levels increase in obesity, insensitivity prevents it from fulfilling its function. Lastly, adiponectin, an anti-inflammatory molecule with protective effects against AD, is decreased in obesity (Letra et al., 2014).

### Connections to Multiple Sclerosis

An association exists between MS and T1DM, however, this is more related to their common origin as autoimmune diseases, and less to an inflammatory environment created by T1DM (Tettey et al., 2015). Links between MS and T2DM are more tenuous. In their review, Tettey et al. (2014) reported evidence for vascular comorbidities in MS, including T2DM. An epidemiological study was unable to find any difference in MS prevalence between diabetic, hypertensive, or hyperlipidemic populations and the general population from 1984 to 2006 (Marrie et al., 2012). More recently, Hou et al. (2017) showed a small, albeit significant, association between T2DM and MS incidence. Another study focusing on MS patients between found that diabetes, obstructive lung disease and hypertension affected clinical outcomes such as walking speed, self-reported disability, and depression (Conway et al., 2017). However, none of these studies provide strong evidence that T2DM increases risk of contracting MS.

As with T2DM, investigations on links between obesity and MS have produced mixed results. The prevalence of obesity amongst MS patients has been found either increased (Slawta et al., 2003), decreased (Markianos et al., 2013; Pinhas-Hamiel et al., 2015), or unchanged (Khurana et al., 2009). A systemic review by Wens et al. (2013) found evidence for increased risk of cardiovascular disease in MS, but the precise connection remains unclear. However, several studies have associated obesity during adolescence with a higher risk of contracting MS (Hedström et al., 2012, 2016). A mendelian randomization study by Mokry et al. (2016) found significant association between genetically induced obesity and MS, with low risk of pleiotropic effects (i.e., the genes in question influence the two conditions through separate pathways).

Possible mechanistic links between obesity and MS has been reviewed elsewhere (Guerrero-García et al., 2016; Palavra et al., 2016). These focus on a few factors, including a possible confounding role played by serum levels of vitamin D, dyslipidemia, and adipokines such as leptin. An increased inflammatory state may be a very important link between obesity, T2DM, and MS. HFDs have been shown to open the CP, which would allow for greater immune sampling and, presumably, greater immune sensitivity (Kanoski et al., 2010). In addition, systemic inflammation would open the BBB, allowing for easier immune penetration (Varatharaj and Galea, 2017). These effects would decrease the threshold required for autoimmune activation, allowing the stimulation of myelin reactive cells.

## Metabolic Syndrome and the Gut Microbiome-Bbb Axis

The gut microbiome has been implicated as an important player in neural health, as well as with obesity and T2DM. Imbalance of the microbiome, known as dysbiosis, has long been known to play a role in the development of metabolic syndrome and obesity (Barlow et al., 2015). For example, germ free (GF) mice gain significantly less weight than specific-pathogen-free (SPF) counterparts, despite higher food intake. Reintroducing bacteria to the GF mice results in further weight gain (Bäckhed et al., 2004). Substantial work has explored the specific contributions of various bacterial phyla to obesity and diabetes, as reviewed elsewhere (Barlow et al., 2015).

More recently, dysbiosis has been shown to be comorbid with neuropathologies including autism spectrum disorder, Parkinson’s disease, MS, and chronic pain (Martin et al., 2018). The microbiota communicate with the brain by interacting with endocrine and enterochromaffin cells, which secrete hormones and neuromodulatory molecules. They also directly signal the brain through peptides, inflammatory molecules, and bacterial metabolites (Martin et al., 2018). Aberration of these signaling pathways leads to neural dysfunction, and gut microbiota profiles have been linked to specific brain profiles, including the size of structures like the hypothalamus, caudate nucleus, and hippocampus (Fernandez-Real et al., 2015; Labus et al., 2017). GF mice showed cognitive deficits, as did *Citrobacter rodentium* infected mice exposed to acute stress (Gareau et al., 2011; Fernandez-Real et al., 2015). In humans, female subjects put on a probiotic milk product for 4 weeks had altered neural activity in affective, viscerosensory, and somatosensory cortices (Tillisch et al., 2013). The microbiota also influences the BBB. GF mice display increased BBB permeability compared to SPF mice, with downregulation of claudin-5 and occludin. Exposure of GF mice to the SPF microbiota decreased BBB permeability and upregulated TJ proteins (Braniste et al., 2014). These findings highlight the importance of the gut microbiota for neural health, but many questions remain unresolved. The microbiota-to-BBB connection has been discovered very recently, and more work is required to elucidate the pathways involved. In addition, most of the work in this area has been done on rodents, and translation to humans remain unclear (Martin et al., 2018).

## The Positive Effects of Inflammation

Despite all the harmful consequences of inflammation, it still serves vitally important functions in the brain that are worth mentioning. Interestingly, many of these functions center around MS, a disease notorious for autoimmune attack driven by inflammation. For example, myelin reactive T cells are normally implicated in neural decline, but when they were injected into rats following partial crush injury of the optic nerve, the rats retained 300% more retinal ganglion cells (Moalem et al., 1999). This beneficial response is specific to anti-myelin T cells. Immune deficient mice given CNS trauma showed improved recovery following treatment with myelin reactive cells, whereas treatment with ovalbumin reactive cells did not have any effect (Yoles et al., 2001). These effects seem to come largely from Th1 and Th2 cells. One study immunizing mice with Th1 and Th2 myelin reactive cells opened the BBB using an antiseptic approach, instead of pertussis toxin, to induce EAE. Instead of a pathological response, Th1 and Th2 both accelerated re-vascularization and healing (Hofstetter et al., 2003), suggesting Th1 and Th2 in a non-inflammatory environment are beneficial for EAE recovery. Another study observed the progression of MS in the mouse brain. Areas with initial oligodendrocyte and myelin loss had high phagocyte count but low B or T cell count. Areas with complete demyelination had high T cell count, an observation apparently connected to oligodendrocyte regeneration (Henderson et al., 2009). Several mechanisms underlying this effect have been explored, and a likely candidate is the release of neurotrophins from activated immune cells that beneficially affect brain recovery. These include compounds like neural growth factor, which modulates B cell proliferation, immunoglobulin production, and cell survival, and brain derived neurotrophic factor, involved in neuronal survival (Hohlfeld et al., 2006; Schwartz and Raposo, 2014).

Such studies contradict the idea that autoreactive T cells are accidental biproducts of failed T cell sorting in the thymus. Indeed, Schwartz and Raposo (2014) defined “protective autoimmunity” as an essential component of CNS health. In their model, an active population of CNS-reactive T cells are essential for proper neuronal development and protection against neurodegenerative diseases such as AD. These cells must be carefully controlled by Tregs, which limit the immune response. While low Treg activity will result in chronic inflammation, high activity of Tregs would lead to neurodegenerative diseases. Such model implies that effective treatment for diseases like MS cannot unselectively suppress the immune system. Instead, it must suppress aspects of the immune system that are destructive, while promoting those that are regenerative.

## Conclusion

Metabolic syndrome and the resulting insulin and leptin resistance and hyperglycemia have pro-inflammatory effects with profound consequences on the BBB. Breakdown of the BBB leads to immune infiltration into the parenchyma and neuronal death. This carries many implications, depending on the brain region affected. Impact on the hypothalamus leads to hormonal disbalance (Boden and Shulman, 2002; Horvath et al., 2010; Jais and Brüning, 2017), damage to the hippocampus leads to cognitive decline (Davidson et al., 2012), and injury to the CP leads to increased immune sensitivity (Kanoski et al., 2010). Many signaling pathways have been implicated in these processes, including VEGF, PKC, RhoA/ROCK, HIF, mTOR, eNOS, AGEs, and miRNA, all of which intersect each other (Fischer et al., 2002; Cirino et al., 2003; Land and Tee, 2007; Yao et al., 2010; Reijerkerk et al., 2013; Shao and Bayraktutan, 2013; Balamurugan, 2016; Serban et al., 2016). Anti-inflammatory drugs target these pathways, but because of the complexity of the picture, it is difficult to predict what side effects they may produce. Given that a certain amount of inflammation is necessary (Moalem et al., 1999), it is vital to carefully select drug targets that will modulate the patient’s immune response in a protective way. Resolving metabolic memory may be one of the more relevant therapeutic approaches, as it causes continued inflammation even after blood glucose levels are resolved (Ceriello, 2012). Furthermore, the effects of glucose on GLUT1 expression levels in the human brain have not been fully resolved. Since hyperglycemia is one of the most important factors causing the conditions described above, understanding why its flux across the barrier increases is essential to understanding and preventing its effects. miRNAs remain a largely unexplored factor in systemic inflammation. Given the increasingly important role miRNAs play in cellular physiology, further study is warranted. Finally, the microbiota has been implicated as a key player in BBB integrity, but remains poorly understood. More work is needed to elucidate this connection.

## Author Contributions

PVD prepared the draft following BL’s instructions. BL chose the theme, edited and corrected the manuscript.

## Conflict of Interest Statement

The authors declare that the research was conducted in the absence of any commercial or financial relationships that could be construed as a potential conflict of interest.
